# Taking a Closer Look at Social Performance in Childhood Social Anxiety Disorder: Biopsychosocial Context Considerations and Effects of Cognitive Behavior Therapy

**DOI:** 10.3390/children9101515

**Published:** 2022-10-04

**Authors:** Julia Asbrand, Brunna Tuschen-Caffier

**Affiliations:** 1 Department of Psychology, Humboldt-Universität zu Berlin, 10099 Berlin, Germany; 2 Institute of Psychology, Albert-Ludwigs-University of Freiburg, 79106 Freiburg, Germany

**Keywords:** social skills, social phobia, treatment, adolescence, psychotherapy

## Abstract

Models of social anxiety disorder (SAD) describe shortfalls in child social performance, whereas empirically, children often show a deficit only in subjective and not objective performance. We examined social performance in relation to possible changes (before and after cognitive behavior therapy [CBT] including social skills training) and to an objective parameter (vocal arousal). Children with SAD were expected to subjectively judge their behavior as less competent than healthy control (HC) children despite a lack of objective differences. Children receiving CBT were expected to show a change in subjective and objective social performance in comparison to children waiting for treatment. Exploratory correlation analyses were used to disentangle the relation between social performance and vocal arousal. One hundred and nineteen children (64 with and 55 without SAD; aged 9–13 years) completed a Trier Social Stress Test (TSST). Children with SAD participated in a second TSST after CBT or waiting. Performance was assessed by self-report and by blinded observers. Vocal arousal was analyzed by audio recording. Children with SAD were objectively assessed as more socially competent than HC children; subjectively, children with SAD showed lower social performance. CBT showed no effect on subjective or objective performance ratings. Vocal arousal did not correlate with social performance. Results need to be considered carefully, as psychometric problems appeared that had not been considered in previous studies. The surprising lack of CBT effects suggests a need to focus on cognitions surrounding social performance. Further, social skills training should not be a standard SAD treatment component but used only if necessary.

## 1. Introduction

Social anxiety disorder (SAD) entails an extensive fear of being embarrassed in social situations and, subsequently, most often an avoidance of these situations [1]. It is highly prevalent in children and youth, with a typical onset during adolescence (up to 9% lifetime prevalence, [2,3]). Despite these facts being uncontroversial, findings on social performance of affected children and youths have been disputed highly. On a theoretical basis, etiological and maintenance models [4,5] stress the role of negative cognitions regarding the individual’s performance (e.g., “Everyone will laugh at me.”) but are more cautious regarding actual performance deficits. They claim the existence of a vicious circle of social performance deficits leading to avoidance of social situations and rising negative cognition about their performance [5]. This is in line with empirical data showing that children and youth with SAD rated their own performance—for example, eye contact, clear speech—as worse than healthy control (HC) children and youths rated their own performance (e.g., [6,7,8,9]). However, controversy marks the findings regarding actual deficits in social performance, that is, observer-rated performance (e.g., [8,10]). For example, [11] found that highly socially anxious children rated their own social performance in a speech task as worse than low socially anxious children rated their own performance. This group difference was also confirmed by objective observers, suggesting an actual social performance deficit and not a cognitive bias. However, as other studies did not find differences in objective ratings (e.g., [6,12]), it is not yet clear if children and youth actually show a social performance deficit or a cognitive bias. Interestingly, Blöte et al. showed that in adolescents with high social anxiety, subjective and objective social performance are not related, which is the case in adolescents with low or medium levels of social anxiety [9]. Thus, a cognitive bias regarding nervousness (e.g., misspeaking), a facet of social performance, and a general tendency to be critical of themselves seem likely in children with SAD [13,14].

The heterogeneous findings lead to the conclusion that other factors might have influenced the previous ambiguous results. For example, trait factors such as age and symptom intensity, but also contextual factors such as the study’s paradigm have been discussed. Alfano et al. reported slight differences in self-reported expected performance during a social-evaluative task between children (<12 years of age) and youth (≥12 years of age [10]). Further, higher levels of anxiety have been found to influence findings in community samples (e.g., [9]), which calls for contrasting high and low levels of social anxiety. Regarding the paradigm, some studies in adults found low levels of objective social performance tended to occur in minimally structured interactions compared to highly structured social interactions [15,16]. Thus, studies using different paradigms should be compared cautiously. To control for possible further influences, highly structured situations should be used when considering potential influences and changes in social performance. Further, as stress seems to be a relevant indicator of social performance deficit, indicators of stress—for example, task difficulty, as indicated by cognitive performance [17,18], and physiological arousal [19,20]—should be considered.

### 1.1. A Stress Model of Social Performance

As indicated above, differences in the set-up of studies might have contributed to the heterogeneous findings. Further, we believe that assessment of social performance might improve with the introduction of a multimethodological background, as previous studies did not focus enough on the psychometric properties of assessment. In our study, therefore, we considered a biopsychosocial stress model of social performance and, thus, included physiological, cognitive, and behavioral aspects.

One possible *physiological* indicator of social performance could be vocal arousal (e.g., a “shaky” voice, talking too softly [19]). This can be analyzed by measuring the fundamental frequency (*f*_0_ [21]), which relates to the frequency of opening and closing of the vocal cords during speech sound production [22]. It can provide information about the emotional arousal of the person speaking [22]. There are two frequently used parameters, *f*_0_ mean, which is the mean of vocal arousal, and *f*_0_ range (*f*_0_ value range), which is the difference between the highest and lowest value of the *f*_0_ and indicates the variability of the voice pitch over an examined period of time (e.g., [23]). Studies suggest that *f*_0_ range increases during emotional arousal and stress [23,24,25]. Preliminary studies with adults, children, and adolescents demonstrated associations of social anxiety and some parameters of vocal arousal [20,21,26]. One of the few studies with children also reported higher average *f*_0_ and more variability in voice pitch during role play in children with SAD compared to children with Asperger’s syndrome, although neither group differed significantly from HC children in this regard [20]. However, vocal arousal has yet to be examined as a stress parameter in relation to social performance.

As mentioned above, the *cognitive* appraisal of one’s own social performance is crucial and rather uncontroversial [6,7,8,9]. An additional component of cognitive facets is cognitive capacity, e.g., correctness in conducting a task, an aspect on which we further report in the Supplementary Materials files. However, the *behavioral* side is ambiguous (e.g., [6,11]). Bringing these aspects together can also shed light on the validity of assessments, as, for example, internal consistency and factor structure are often not reported.

Finally, one possible path to further zoom in on social performance is an experimental manipulation aiming to decrease SAD-relevant stress levels in general or change social performance deficits in particular. A clinical change of SAD symptoms has been achieved by treatments that have stressed the relevance of including social performance or social skills training (e.g., [27,28]). More recently, the combination of the gold-standard treatment—cognitive behavior therapy (CBT)—and social skills training has been recommended [29]. However, although these interventions focused on SAD symptoms in general or on social performance deficits, no study has yet used an intervention to disentangle the contradictory findings of subjective and objective social performance in SAD. Thus, subjective and objective social performance should be assessed in a standardized social stress test (i.e., Trier Social Stress Test for Children [TSST-C] [30]) before and after CBT. Given the above-mentioned influence factors, the sample should be carefully chosen regarding age (an age span of preadolescent children of 9–13 years) and symptom severity (no difference between experimental groups). We targeted the overall strategy of assessment of social performance regarding psychometric properties and biopsychosocial considerations in a highly controlled set-up, and those concerning the potential of change by including an intervention.

### 1.2. The Current Study

We began with a thorough psychometric analysis of all assessments. We then expected children with SAD to rate their own behavior as less competent than HC children would rate their own behavior (cognitive factor) despite no objective differences (behavioral factor). We expected children receiving CBT to show a change in subjective social performance ratings as well as a change in objective social performance ratings compared to children in a wait-list control (WLC) group (social performance in relation to CBT). Finally, to achieve a closer look at social performance, explorative correlation analyses, including behavioral aspects (objective ratings of social performance), cognitive aspects (subjective ratings of social performance), and physiological aspects (vocal arousal including *f*_0_ mean and *f*_0_ range), were performed.

## 2. Materials and Methods

### 2.1. Study Design

The project uses a cross-sectional study design (comparing children with SAD and HC children on cognitive, behavioral, and psychophysiological variables) combined with a subsequent randomized controlled trial including treatment for children with SAD. The project’s main focus was on CBT as an experimental manipulation. Prior to recruitment, we registered eligibility criteria with the German Research Foundation which were not changed during the study. This study was part of a larger project that consisted of experimental studies related to research questions of visual attention allocation or psychophysiological processes under (social) stress and it also aimed to measure treatment success by including several outcome variables (state anxiety, negative cognitions, physiological arousal, perception of and worry about physiological symptoms, perception of academic performance, negative postevent processing, parental cognitions, parental fear of negative child evaluation, and related treatment outcome predictions). We reported the majority of the a priori-defined outcome variables and secondary outcomes in earlier papers (treatment outcome [31]; changes in postevent processing based on treatment [32]; stability of the cortisol response despite treatment [33], physiological arousal [34] and perception of physiological arousal treatment [35]). To ensure maximal transparency, all articles include cross-references to other reports on measures used to investigate potential treatment-related effects. Outcomes of self-report of social anxiety have been reported elsewhere as a self-perception assessment [31]. However, because of limitations on length and foci, detailed findings on social performance in relation to observer-rated performance have not yet been reported in its entirety.

The current study reports secondary outcome variables relating to objective and subjective social performance. A power analysis was conducted using G*Power version 3.1.9.7 [36]. We applied both a repeated measures analysis for two analyses (two groups, one dependent variable, source as repeated measure; two groups, one dependent variable, source and time as repeated measures) and a multiple correlation analysis. The sample size for the current study, based on a small-to-medium effect [8] and power of (1 − β) = 0.80, was set for the largest necessary sample size at *n* = 90 (each group *n* = 45). As the study was part of a larger research project requiring a larger sample size of *n* = 110, all children were included to increase power. As this project was conducted with a larger focus, this Method section has been reported before in a similar fashion (e.g., [31]). Data cannot be shared publicly as this is not explicitly included in the informed consent by participants and the mental health data of children is particularly sensitive. Deidentified participant data with annotations will be made available to other researchers upon reasonable request (e.g., meta-analysis) by the first author.

### 2.2. Participants

**Figure 1:**
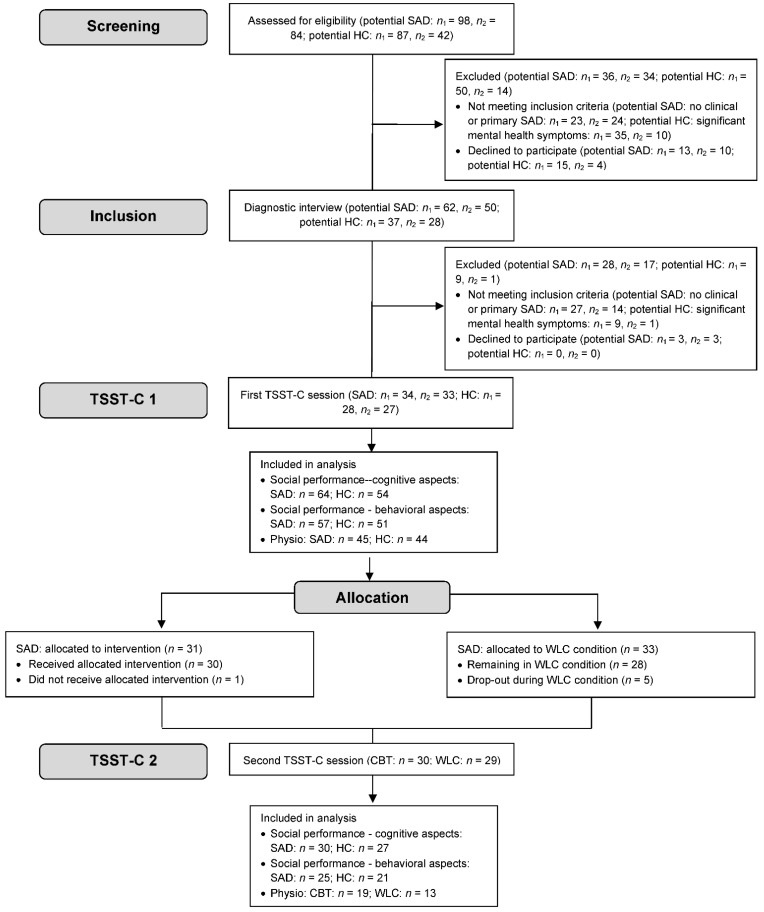
Flowchart of study participants. Note: missing data were mostly based on technical difficulties, that is, no video/audio recording. *n*_1_ = Center 1, *n*_2_ = Center 2; CBT = cognitive behavior therapy; HC = healthy control; SAD = social anxiety disorder; TSST-C = Trier Social Stress Test for Children; WLC = wait-list control.

Families with anxious and non-anxious children (9 to 13 years) were approached through advertisements in schools, medical facilities, and newspaper articles in two midsized German cities from January 2012 to November 2013 until the targeted sample size had been reached (for an overview see Figure 1). No harms were reported. The treatment trial was part of a larger project, which is presented elsewhere [31]. In compensation for participation in the laboratory study, parents received €35, and children €25 in vouchers. An independent ethics committee (ethics committee of the German Society for Psychology [DGPs]) granted ethical approval for this study. Participating children and their caregivers gave oral and written informed consent.

Children were included in the SAD group if they showed SAD as primary diagnosis; children were included in the HC group if they reported no current or lifetime diagnosis of a mental disorder. Health problems or medication that could have interfered with psychophysiological assessment (e.g., asthma, cardiac arrhythmia, and methylphenidate) led to exclusion. Social Anxiety Scale for Children–Revised (SASC-R; [37]) scores exceeded suggested cutoffs for clinically relevant SAD.

The study was conducted at two German universities. All analyses first considered site differences, which were nonexistent. Participants with SAD were randomized to the CBT or the WLC group. We used a concealed randomization in each center by the other center, which was based on subject codes, as soon as there were enough participants for one experimental and one WLC allocation.

### 2.3. Procedure

A telephone screening for anxiety symptoms preceded the diagnostic session for eligible children and their parents (see flowchart in Figure 1). Even though we invited both mothers and fathers to participate, data was mostly only available from mothers, which is why we refer to mothers whenever parent reports are needed. Diagnoses of SAD and comorbid disorders *Diagnostic and Statistical Manual of Mental Disorders*, 4th ed., [38] were reached by combining individual structured clinical interviews with both the child and a parent, performed separately, using the German-language Diagnostic Interview for Mental Disorders in Children and Adolescents (Kinder-DIPS; [39]). It was conducted by trained graduate student interviewers and supervised by an experienced clinical psychologist. The Kinder-DIPS provides a validated interview for the most frequent mental disorders in children and youth, i.e., the Kinder-DIPS shows adequate interrater reliability (87% for anxiety disorders), good retest reliability, and successful validation with disorder-specific questionnaires [39]. Further, children and parents completed online questionnaires on sociodemographic data, anxiety symptoms, and general psychopathology. In conclusion of the diagnostic assessment, 67 children fulfilled the inclusion criterion of a primary diagnosis of SAD; 55 children were included in the HC group.

**Figure 2:**
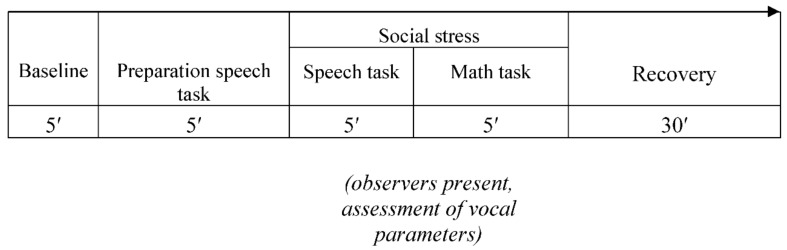
Overall procedure including the Trier Social Stress Test for Children depicted in minutes (TSST-C). The same procedure was followed before and after treatment or waiting.

After the diagnostic interviews, children completed a first laboratory session where they were given the TSST-C (TSST-C 1; [30]), consisting of a speech and a math task in front of two observers (see Figure 2; cf. [31]). The observers were trained to provide only neutral verbal and nonverbal feedback. After the main stress task (story and math task), children were asked to rate their social performance (subjective performance rating). After participating in a 12-week CBT program or waiting without treatment, all children performed a parallel version of the first testing session (TSST-C 2). The TSST-C reliably induces social anxiety in all children.

### 2.4. Psychometric Measures

The SASC-R [37] measures self- and parent-reported symptoms of social anxiety in children (18 items, e.g., “I only talk to boys and girls I know well”). Total scores range from 18 to 90. Each item can be answered by child or parent, resp., using a 5-point Likert-type scale ranging from 1 (*not at all*) to 5 (*all the time*). Both test–retest reliability (0.67) and internal consistency (0.76) are satisfactory. The internal consistency of the SASC-R in the current sample was excellent (child report: α = 0.95, mother report: α = 0.97).

### 2.5. Cognitive Measure: Social Performance Self-Report

The Performance Questionnaire-Child (PQ-C; [7]) is a nine-item instrument that assesses three aspects of self-rated social performance. In addition to subscales measuring nervous behaviors (e.g., “Did you stumble over your words?”) and global impression (e.g., “How friendly did you look?”), a microbehaviors subscale assesses social performance areas that are typically taught in social skills training programs (e.g., “How much did you look at the person you were talking to?”). All nine items were scored on a 4-point scale ranging from 0 (not very much) to 3 (very much), with a potential range of 0 to 27 for the overall scale. The current study used the German translation [8]. A more positive evaluation of social performance is indicated by higher values. Psychometric properties are reported in the Results section.

### 2.6. Behavioral Measures: Social Performance Other-Report

The PQ-O (Performance Questionnaire-Observer [7]) is completely parallel to the PQ-C. We also used the German translation with nine items relating to three scales [8]. A more positive evaluation of social performance is indicated by higher values. Objective social performance was rated by advanced graduate students in clinical psychology who were blind to children’s diagnostic status. All coders received 1 day of training on the use of the PQ, with multiple training videos under supervision of the first author.

The TSST-C 1 was rated by all coders (Coder 1: 100%, Coder 2: 100%, Coder 3: 65%). A first evaluation was conducted with two coders. As the results were surprising (see below), an additional coder was brought in to confirm the ratings. As all three coders showed high agreement, confirming the first result, all coders were included in the final analyses as presented here. The TSST-C 2 was rated by two coders (Coder 1: 100%, Coder 2: 10%). Final scores were calculated by averaging individual scores if more than one score was available. Psychometric properties are reported in the Results section. Due to technical difficulties, some TSST-C sessions were not recorded and could therefore not be evaluated objectively (n_SAD_ = 6, n_HC_ = 4).

### 2.7. Physiological Measures: Vocal Arousal

We assessed *f*_0_ during the story and math part of the TSST-C. We included both *f*_0_ range and *f*_0_ mean. Prior to calculating *f*_0_, the audio recordings were checked for background noise and other artifacts (experimenter speaking; long breaks, etc.). Our analysis included the normal range of speech by setting the floor at 75 Hz and ceiling at 300 Hz [40]. Minimum and maximum *f*_0_ values and *f*_0_ mean were generated by using Praat, a free voice analysis program (Version 6.0.46; [41]). The *f*_0_ range was calculated by subtracting each participant’s minimum *f*_0_ from their maximum *f*_0_. Outliers (*SD* ± 3) were not included in the analyses.

### 2.8. Treatment

Treatment was conducted as a standard exposure-based CBT group treatment by trained graduate students and clinical psychologists that was evaluated simultaneously [31]. The CBT targeted maladaptive cognitions, social competence and avoidance using an emphasis on exposure. Sessions entailed 100 min (including a 10-min break) in groups of five to seven children. The training consisted of 12 sessions covering five modules: psychoeducation, cognitive restructuring, social skills training, exposure, and relapse prevention. To ensure a transfer into everyday life, therapists further encouraged the use of newly developed skills outside of treatment (for more information see treatment manual; [42].

### 2.9. Statistical Analysis

Building on psychometric considerations, we first conduced an analysis of the intraclass correlations and internal consistency of the self- and other-reported social performance. Social performance in relation to SAD was analyzed using a repeated measures analysis of variance (ANOVA) with source of rating (subjective, objective) and group (SAD, HC) as independent variables, and social performance as the dependent variable. Treatment effects (social performance in relation to CBT) were analyzed using a similar set-up with the addition of the repeated measure of session (TSST-C 1, TSST-C 2). Finally, exploratory correlation analyses were conducted between cognitive factors (subjective social performance), behavioral factors (objective social performance), and physiological arousal (*f*_0_ range, *f*_0_ mean). This led to a multiple correlation analysis before treatment including all children and a repeated measures correlation including all children in the SAD group after treatment. Analyses were conducted using IBM SPSS statistics version 25. Missing data were mostly based on technical difficulties, that is, no video/audio recording.

## 3. Results

### 3.1. Participant Characteristics

Demographics and psychometric measures are reported in Table 1. The groups did not differ in age, type of school, or any of the disorder-specific measures.

Children in the CBT and WLC groups did not differ in sociodemographic or psychopathological variables (see Table 2).

### 3.2. Psychometric Considerations of Self- and Other-Report of Social Performance

An intraclass correlation (ICC) was conducted to examine interrater agreement on the PQ. The correlation between the coders’ scores was good for all scales during the TSST-C 1 (micro behaviors: ICC = 0.834; nervousness: ICC = 0.788; global impression: ICC = 0.859) as well as for the overall score (ICC = 0.871). Similarly, correlation between coders was high for two subscales during the TSST-C 2 (micro behaviors: ICC = 0.836; global impression: ICC = 0.889) as well as for the overall score (ICC = 0.729). However, the ICC for nervousness was inadequate (ICC = 0.225).

Although the questionnaire has shown acceptable internal consistency in one previous German study [8], overall only some of the previous studies using the PQ reported values regarding internal consistency [6,44]. However, these reports usually did not distinguish between self- and other-reported data. We therefore opted to use only measures showing adequate evidence (α > 0.60) of internal consistency for both scales (microbehaviors: α_self_ = 0.054, α_other_ = 0.290; nervousness: α_self_ = 0.609, α_other_ = 0.427; global impression: α_self_ = 0.726, α_other_ = 0.681). To ensure that our results were not biased, we applied an additional measure of internal consistency, namely McDonald’s omega [45]. It resulted in the following internal consistencies: microbehaviors: ω_self_ = 0.126, CI [0.028, 0.524], ω_other_ = 0.401, CI [−0.425, 1.226]; nervousness: ω_self_ = 0.622, CI [0.500, 0.744], ω_other_ = 0.552, CI [0.427, 0.685]; global impression: ω_self_ = 0.740, CI [0.658, 0.821], ω_other_ = 0.708, CI [0.608, 0.908]. As a rule of thumb, values above 0.70 are adequate in research [46]. Further, for microbehaviors (self-report) and nervousness (other-report), the omega calculation with a confirmatory factor analysis failed and a principal factor analysis was used. Thus, both values and the failed CFA point to a lack of validity for microbehaivors and nervousness. Thus, only global impression was used in the current analysis, hence leading to a potential range of 0 to 9.

### 3.3. Social Performance

**Figure 3:**
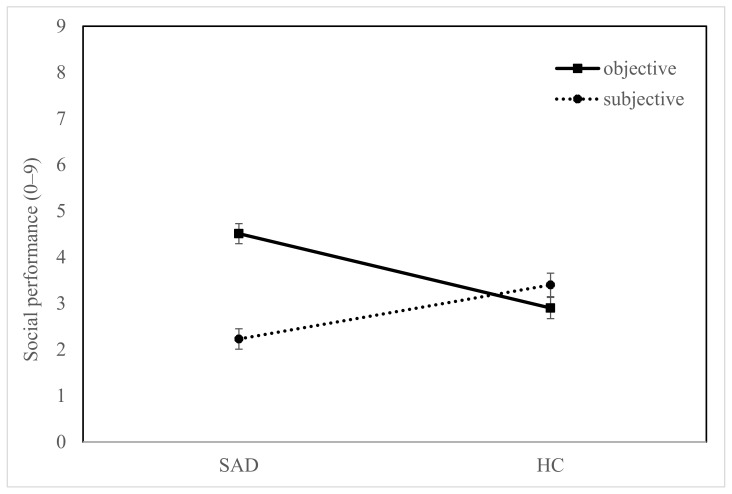
Social performance differences between children with SAD and HC and objective and subjective coders.

The repeated measures ANOVA revealed a significant difference between objective and subjective sources, *F*(1105) = 11.99, *p* = 0.001, η_p_^2^ = 0.102, and a significant interaction between source and group, *F*(1105) = 27.50, *p* < 0.001, η_p_^2^ = 0.208, but no difference between groups, *F*(1105) = 3.61, *p* = 0.239, η_p_^2^ = 0.013 (see Figure 3). The post hoc *t* tests for independent samples showed—as expected—a lower social performance score in children with SAD, *M* = 2.23, *SD* = 1.77, compared to HC children, *M* = 3.40, *SD* = 1.87, *t* (116) = −3.50, *p* = 0.001, *d* = 0.64, CI [0.27, 1.02]. However, objective coders rated children with SAD, *M* = 4.51, *SD* = 1.64, to be more socially competent than HC children, *M* = 2.90, *SD* = 1.64, *t*(106) = 5.11, *p* < 0.001, *d* = −0.98, CI [−1.38, −0.58]. This surprising result was supported by all coders (see interrater reliability, above).

In sum, both subjective and objective ratings show a significant difference between children with and without SAD in social performance. However, objective ratings show a *better* performance in children with SAD, whereas children with SAD evaluated their performance as *worse* compared to HC children’s self-ratings.

### 3.4. Social Performance in Relation to CBT

The comparison of social performance before and after CBT or waiting revealed a significant effect of source, *F*(1,42) = 44.72, *p* < 0.001, η_p_^2^ = 0.516, session, *F*(1,42) = 16.69, *p* < 0.001, η_p_^2^ = 0.284, and Source × Session, *F*(1,42) = 5.442, *p* = 0.025, η_p_^2^ = 0.115. No other effects reached significance, *F*s < 3.22, *p*s > 0.079. Thus, as no effect relevant to the hypotheses appeared, that is, no group effects, no post hoc analyses were conducted.

### 3.5. Biopsychosocial Considerations of Social Performance

During the TSST-C 1, significant correlations appeared among cognitive and behavioral aspects (subjective and objective social performance), *r* = −0.234, *p* = 0.015 (Table 3). Finally, trait social anxiety related negatively to subjective social performance, *r* = −0.415, *p* < 0.001, and positively to objective social performance, *r* = 0.400, *p* < 0.001. That is, higher trait anxiety was related to lower cognitive appraisal of social performance but to higher behavioral levels of social performance.

For the second TSST-C (after CBT or waiting), we chose a repeated measures approach. No significant correlations remained (see Table 4).

## 4. Discussion

The purpose of the current study was to shed light on the possible challenges in assessment of social performance as well as its potential dimensions. Children with SAD, compared to HC children, were expected to rate their behavior as less competent despite there being no objective differences. Interestingly, both subjective and objective ratings showed a significant difference between children with and without SAD in social performance. However, objective ratings showed a *better* performance in children with SAD, whereas the self-rated social performance of children with SAD was *worse* than the self-rated social performance of HC children. Further, an effect of CBT on subjective and objective social performance was expected but not found. Finally, a biopsychosocial stress model of social performance including behavioral, cognitive, and physiological aspects was exploratively examined. Here we could show that correlations appeared between behavioral and cognitive factors as well as in relation to trait anxiety. Physiological factors were not significant.

### 4.1. The Relevance of Subjective and Objective Social Performance in Childhood SAD

A more negative self-rated social performance in children with SAD was expected and would be in line not only with theoretical models [4] but also with empirical findings [6,7,8,9,14]. The unexpected positive bias in objective ratings was checked repeatedly (see Materials and Methods) and found to be stable. As previous research has been inconclusive regarding whether all children with SAD suffer from a social performance deficit, our findings might suggest that children with SAD can perform well in a social performance situation.

Our findings might be attributable to specifics of our sample (i.e., highly skilled children with SAD). Although they were recruited on two different sites and meticulously diagnosed, a random selection of highly socially skilled children might have occurred, as low social performance was not a selection criterion. Further, effects might have occurred because of activation of additional resources due to facing the situation as a social challenge instead of a threat [47]. Possibly, children with SAD put more effort into tasks such as the TSST-C as they see a higher relevance for themselves. Interestingly—and favorable to the children with SAD—they have the resources to perform on a high level. As suggested by Hase et al., we used a rigorous protocol that induces stress not only in children with SAD but also in HC children and combined several factors (i.e., behavioral, cognitive, physiological [47]). This once again refers to the relevance of subjective and objective stress levels, leading to a holistic picture of social performance.

It might be interesting to take a closer look at the coders, who were all young adults. Three sets of coders were highly trained and blinded to the children’s diagnosis and treatment status, leading to high agreement among repeated assessments using different coders. Although this underlines the stability of findings and stresses the relevance of context, peer coders might be a future alternative: They have been found to be more critical and possibly provide a more ecologically valid picture [48] and could therefore be used in future studies. An additional analysis of subgroups of children with SAD (i.e., children with objectively high vs. low social performance levels; [9]) might be an interesting approach in future research; it was not applicable in our study because of our sample size. Finally, our surprising finding might further stress the importance of branching out into other aspects of social performance and broadening the concept of assessment to include a biopsychosocial model.

Overall, the negative self-rating compared to a positive other-rating in children with SAD once again directly stresses the existence of a cognitive bias. This is supported by the finding of a negative correlation between subjective and objective performance; that is, the higher the child’s performance on an objective level, the lower the performance and thus more critical is the child. This suggests that therapists should assess very carefully at the beginning of treatment if a social performance deficit exists or—what is more likely—only a cognitive bias, which would then lead to a focus on cognitions instead of social skills training. Focusing only on the latter could lead to the adverse effect of sustaining negative beliefs [7].

Interestingly, no differences appeared as a result of CBT. Explanations for a lack of effect for the behavioral side might stem from methodological issues such as a ceiling effect before treatment in the objective assessment of the behavior domain but also from the limited sample size, which might have been too small to detect small effects. This was even more surprising regarding cognitive appraisal of social performance: starting from low values before treatment, an increase would have been expected. This might be related to the high stress level that the TSST-C typically produces. Overall, this was the first study targeting the potential of CBT as a mechanism of change and could suggest that a focus on positive cognitions and self-appraisal in interaction tasks might be crucial [32].

### 4.2. Implications for a Biopsychosocial Stress Model of Social Performance

The exploratory analysis of relations between cognition, behavior, and physiology interestingly showed a relation only between cognitive (self-report) and behavioral (other-report) aspects of social performance. Even more astoundingly, a higher self-report was related to a lower other-report. This might indicate a bias in both groups of children: that is, HC children may have rated their own performance as more positive than it was and children with SAD may have rated their performance as more negative than it was. Although the latter has been discussed before, the first interpretation might be in line with a positivity bias often found in depression (e.g., [49]). The lack of a physiological effect is disappointing but not overall surprising. Although there might be a physiological aspect of social performance [19], it has been suggested that it is more the perception of physiological arousal than actual physiological arousal that is important in SAD [50]. As this has not been discussed before, we strongly recommend further studies of multimethodological perspectives on social performance.

Some limitations should be considered, but also should possible strengths. First, objective behavior analysis was difficult in some cases as a few children did not speak at all. Coders were advised to refer to the PQ and leave out some questions (e.g., did the child misspeak). However, we stressed the selection of a questionnaire and procedure that have been used in a variety of studies and research groups, which emphasizes the validity of the set-up [6,8,51]. Second, there was no a priori hypothesis for a biopsychosocial model including vocal parameters before data collection started. However, as the study covered a wide range of parameters and included a video analysis, vocal parameters were included later and proved to provide an additional perspective. Third, as mentioned above, to provide a more ecologically valid analysis, further studies could use peer coders instead of or in addition to adult coders. Fourth, the current study faced methodological challenges despite a rigid design, multiple codings, and the use of established instruments. We present these challenges openly and hope to encourage further discussion and focus on thorough psychometric testing. Fifth, we cannot rule out an interference of the socio-economic background of our participants. As it often is the case in university city samples, income was on the higher side. However, groups did not differ regarding these parameters. Finally, small effects for treatment could not be found given the current sample size. As mentioned above, this is a secondary analysis in a larger project and, therefore, considerations regarding a higher probability of missing data for social performance could not be included. We experienced some—even though not major—problems with both audio and video recording and could therefore not include all children’s performances. We appreciate that this might lead to power problems and—potentially—a bias. We therefore further recommend seeing the current study as an exploratory further step in understanding social performance also on a methodological level, and ask the following questions: are the current measures for assessing social performance from different perspectives valid? Can we change social performance on a behavioral level, or just the cognitive appraisal?

The current study has added to a complex and heterogenous body of research on social performance in childhood SAD. In line with most research [6,7,8,9], subjective ratings of social performance were lower in children with SAD than in HC children. As objective ratings were found to be more positive in children with SAD, future research on further objective parameters such as vocal arousal is warranted to provide a more conclusive picture of social performance. The lack of an effect of CBT warrants both further research and a possible stronger focus on performance-related cognitions during CBT. As the idea of biopsychosocial stress factors as contextual influences on social performance was introduced in this study, additional research regarding different domains could complete the complex picture of social performance.

## Figures and Tables

**Table 1:** Participant Characteristics: Social Anxiety Disorder Versus Healthy Control Group.

Variable	Group		Statistics
	Social Anxiety Disorder (SAD)	Healthy Control (HC)	
*N*	67	55	
Age (in years) ^a^	11.3 (1.4)	11.3 (1.4)	*t* (117) = 0.06, n.s.
% female	63.6	60.0	χ^2^ (1) = 0.17, n.s.
Mean SPAI-C *(SD)*	23.3 (9.03)	4.2 (5.4)	*t* (117) = −13.71 ***, d = −2.71, CI [−3.28, −2.14]
Mean SASC-R ^a,b^ (child report) *(SD)*	49.4 (13.0)	28.2 (8.7)	*t* (117) = 10.59 ***, d = −2.03, CI [−2.54, −1.52]
Mean SASC-R ^a,c^ (mother report) *(SD)*	60.9 (11.0)	28.4 (6.9)	*T* (114) = 19.34 ***, d = −3.44, CI [−4.10, −2.78]
Monthly income (%)			χ^2^ (8) = 11.42, n.s.
NA	0	1.3	
<€1000	0	5.9	
<€1500	1.9	7.4	
<€2000	11.1	8.8	
<€3000	35.2	32.4	
<€4000	14.8	16.2	
<€5000	14.8	20.6	
>€5000	22.2	7.4	
State anxiety during TSST-C (before treatment) ^a^	6.6 (2.8)	4.5 (2.9)	*t* (117) = 4.05 ***, d = −0,75, CI [−1.12, −0,37]

Note. NA = Not available; SASC-R = Social Anxiety Scale for Children–Revised (cutoffs: 50 for boys, 54 for girls; [37]), SPAI-C (Social Phobia and Anxiety Inventory for Children [43]); ^a^ Mean (*SD*). ^b^ Missing data: *n*_SAD_ = 3, *n*_HC_ = 0. ^c^ Missing data: *n*_SAD_ = 4, *n*_HC_ = 2. *** *p* ≤ 0.001; n.s. = not significant.

**Table 2:** Participant Characteristics: Treatment Versus Wait-List Control Group.

Variable	Group	
	Treatment (CBT)	Wait-List Control (WLC)
*N*	31	33
Age (in years) ^a^	11.5 (1.4)	11.2 (1.3)
% female	51.6	67.6
Mean SPAI-C *(SD)*	21.9 (10.2)	23.7 (7.74)
Mean SASC-R ^a,b^ (child report) *(SD)*	49.3 (14.0)	49.6 (12.3)
Mean SASC-R ^a,c^ (mother report) *(SD)*	60.5 (12.5)	61.3 (9.5)
Monthly income (%)		
NA	3.2	0
<€1000	6.5	5.6
<€1500	9.7	5.6
<€2000	6.5	8.3
<€3000	41.9	23.7
<€4000	16.1	16.7
<€5000	9.7	30.6
>€5000	6.5	8.3
State anxiety during TSST-C (before treatment) ^a^	6.7 (2.9)	6.6 (2.8)

Note. CBT = cognitive behavior therapy; NA = not available; n.s. = not significant; SASC-R = Social Anxiety Scale for Children–Revised (cutoffs: 50 for boys, 54 for girls [37]); SPAI-C (Social Phobia and Anxiety Inventory for Children; [43]). ^a^ Mean (*SD*). ^b^ Missing data: *n*_CBT_ = 0, *n*_WLC_ = 0. ^c^ Missing data: *n*_CBT_ = 0, *n*_WLC_ = 1.

**Table 3:** Means, Standard Deviations, and Pearson Correlations for Variables Before CBT or Waiting (*n* = 118).

Variable		*M*	*SD*	1.1	2.1	3.1	3.2
Social performance (cognitive)	1.1 Self-report	2.77	1.90				
Social performance (behavioral)	2.1 Other-report	3.75	1.82	−0.23 *			
Physiological arousal	3.1 *f*_0_ mean	218.6	25.58	−0.11	−0.19		
	3.2 *f*_0_ range	42.9	10.19	−0.13	−0.06	−0.06	
Trait social anxiety	4.1 SPAIC	13.81	12.14	−0.42 ***	0.40 ***	−0.09	0.05

Note. CBT = cognitive behavior therapy; *f*_0_ = fundamental frequency; SPAI-C (Social Phobia and Anxiety Inventory for Children; 38). * *p* < 0.05, *** *p* < 0.001.

**Table 4:** Means, Standard Deviation and Pearson Correlations for Variables After CBT or Waiting (*n* = 36).

Variable		*M*	*SD*	1.1	2.1	3.1	3.2
Social performance 2 (cognitive)	1.1 Subjective	2.39	1.91				
Social performance 2 (behavioral)	2.1 Objective	5.52	1.82	−0.14			
Physiological arousal 2	3.1 *f*_0_ mean	206.92	25.47	−0.09	0.08		
	3.2 *f*_0_ range	39.53	11.27	−0.28	−0.11	−0.18	
Trait social anxiety	4.1 SPAIK	18.79	8.85	−0.20	−0.19	−0.31	0.21

Note. CBT = cognitive behavior therapy; *f*_0_ = fundamental frequency; SPAI-C (Social Phobia and Anxiety Inventory for Children [43]).

## Data Availability

Data cannot be shared publicly as this is not explicitly included in the informed consent by participants and the mental health data of children is particularly sensitive. Deidentified participant data with annotations will be made available to other researchers upon reasonable request (e.g., meta-analysis) by the first author.

## Supplementary Materials

The following supporting information can be downloaded at: https://www.mdpi.com/article/10.3390/children9101515/s1, Supplemental Materials file: Additional cognitive aspects.
