# Supplementary material for: Taking a Closer Look at Social Performance in Childhood Social Anxiety Disorder: Biopsychosocial Context Considerations and Effects of Cognitive Behavior Therapy
Source: Children (Basel). 2022 Oct 4;9(10):1515. doi: 10.3390/children9101515 (PMC9600406; doi:10.3390/children9101515)
Supplement: Supplementary file 1 [file children-09-01515-s001.zip › children-1874658-supplementary.pdf]

Supplement: Additional cognitive aspects

In addition to cognitions about a situation, cognitive performance is crucial to successfully mastering a situation. Cognitive performance relates to components of task performance such as mental operations, information decoding, and encoding as well as memory processes (1). There is currently a large body of evidence pointing to deficits in cognitive performance during anxiety-evoking situations in children and adults with high levels of anxiety (1,2), which has even been translated into intervention recommendations (3). However, specific research on social anxiety disorder (SAD) in children and youth is scarce. Two studies in a preadolescent sample with SAD (8-12 years old; (4); 7-14 years old; (5) did not show decreased levels of cognitive performance in comparison to healthy control (HC) children. However, cognitive models (6) have convincingly claimed that cognitive resources in social-evaluative situations are mainly allocated to attention to potential external threats and monitoring internal cues, leaving only limited resources for proper completion of the task. These claims were verified in samples of adults with SAD (e.g., (7). Even if there were no differences to be found in preadolescent children, the relation of cognitive to behavioral and physiological performance seems worth understanding.

Methods: Arithmetic Capacity

Arithmetic capacity (operationalized by absolute steps in calculation and error rate) as a cognitive factor related to social performance was included post hoc in an exploratory manner. The math task was analyzed by an additional graduate student, who was again blind to the diagnostic status of the children. Absolute steps in calculation, correct calculations, and errors were counted. An error rate was calculated by dividing errors by absolute steps in the calculation.

In this supplement, we report exploratory correlation analyses that were conducted between cognitive factors (subjective social performance), behavioral factors (objective social performance), and physiological arousal (fundamental frequency [f_0] range, f_0 mean) and arithmetic capacity (absolute steps in calculation, error rate).

Results

During the TSST-C 1, additional significant correlations appeared among cognitive aspects (absolute steps in calculation and error rate), $r = -.337$, $p < .001$. Regarding other domains, an additional correlation was found only between physiological aspects (f_0 range) and error rate, $r = -.225$, $p = .037$. That is, a higher f_0 range related to a lower error rate (see Table S1). Finally, trait social anxiety was positively related to error rate, $r = -.254$, $p = .018$. After treatment, no correlation remained for the additional cognitive aspects (see Table S2).

Table S1

*Means, Standard Deviations, and Pearson Correlations for Variables Before CBT or Waiting (*N *= 118) Supplemented By Additional Cognitive Aspects*

Variable		*M*	*SD*	1.1	2.1	3.1	3.2	4.1	4.2
Social performance (cognitive)	1.1 Self-report	2.77	1.90						
Social performance (behavioral)	2.1 Other-report	3.75	1.82	-.234*					
Physiological arousal	3.1 f_0 mean	218.6	25.58	-.109	-.193				
	3.2 f_0 range	42.9	10.19	.126	-.067	-.060			
Additional cognitive aspects	4.1 Absolute steps in calculation	34.10	19.77	.114	-.110	-.050	.123		
	4.2 Error rate	0.21	0.21	-.070	.081	-.088	-.225*	-.337***	
Trait social anxiety	5.1 SPAIC	13.81	12.14	-.415***	.400***	-.088	.053	-.82	.254*

Note. CBT = Cognitive behavior therapy; f_0 = fundamental frequency; SPAI-C (Social Phobia and Anxiety Inventory for Children (8)

*p < .05. ***p <.001.

Table S2

*Means, Standard Deviation and Pearson Correlations for Variables After CBT or Waiting (*N *= 36) Supplemented by Additional Cognitive Aspects*

Variable		*M*	*SD*	1.1	2.1	3.1	3.2	4.1	4.2
Social performance 2 (cognitive)	1.1 Subjective	2.39	1.91						
Social performance 2 (behavioral)	2.1 Objective	5.52	1.82	-.175					
Physiological arousal 2	3.1 f_0 mean	206.92	25.47	-.016	-.079				
	3.2 f_0 range	39.53	11.27	.100	-.009	.141			
Additional cognitive aspects 2	4.1 Absolute steps in calculation	33.31	18.41	.153	-.287	.067	.333		
	4.2 Error rate	0.16	0.21	.180	-.004	-.152	.289	-.204	
Trait social anxiety	5.1 SPAIK	18.79	8.85	-.210	-.207	-.458*	-.450*	.035	-.131

Note. CBT = Cognitive behavior therapy; f_0 = fundamental frequency; SPAI-C (Social Phobia and Anxiety Inventory for Children (8)

*p < .05.

Discussion

A correlation at baseline before treatment was found between cognition (error rate) and physiology (f_0 range). Interestingly, a lower f_0 range (physiology) was related to a higher error rate (error rate). The f_0 range has been shown to be an indicator of negative emotions and distress (9,10), which in turn have been shown to negatively affect cognitive performance or cognitive load (11,12). Thus, vocal arousal seems to be a valid physiological parameter and potentially very essential in social anxiety (13). However, these parameters as potential markers for stress did not lead to clarification in relation to social performance. Thus, extended research on a broad level of domains regarding social performance is necessary.

References

1. Ashcraft MH, Kirk EP. The relationships among working memory, math anxiety, and performance. J Exp Psychol Gen. 2001;130(2):224–37.
2. Owens M, Stevenson J, Norgate R, Hadwin JA. Processing efficiency theory in children: Working memory as a mediator between trait anxiety and academic performance. Anxiety, Stress Coping. 2008;21(4):417–30.
3. Manassis K. Cognitive findings in childhood anxiety: Translations for clinical practice. Transl Neurosci [Internet]. 2013;4(1):88–95.
4. Krämer M, Schmitz J, Heinrichs N, Tuschen-Caffier B. Self-evaluation, social and cognitive performance in children with social phobia. J Exp Psychopathol. 2011;2(4):586–600.
5. Spence SH, Donovan C, Brechman-Toussaint ML. Social skills, social outcomes, and cognitive features of childhood social phobia. J Abnorm Psychol. 1999;108(2):211–21.
6. Rapee RM, Heimberg RG. A cognitive-behavioral model of anxiety in social phobia. Behav Res Ther. 1997;35(8):741–56.
7. Rapee RM, Abbott MJ. Modelling relationships between cognitive variables during and

following public speaking in participants with social phobia. Behav Res Ther [Internet]. 2007 Dec [cited 2012 Aug 22];45(12):2977–89.

8. Beidel DC, Turner SM, Hamlin K, Morris TL. The Social Phobia and Anxiety Inventory for Children (SPAI-C): external and discriminative validity. Behav Ther. 2001;31(1):75–87.

9. Weusthoff S, Baucom BR, Hahlweg K. Fundamental frequency during couple conflict: An analysis of physiological, behavioral, and sex-linked information encoded in vocal expression. J Fam Psychol. 2013;27(2):212–20.

10. Juslin PN, Scherer KR. Vocal expression of affect. In: Harrigan JA, Rosenthal R, Scherer KR, editors. The new handbook of methods in nonverbal behavior research. Oxford, UK: Oxford University Press; 2005. p. 65–73.

11. Plass JL, Kalyuga S. Four ways of considering emotion in cognitive load theory. Educ Psychol Rev [Internet]. 2019;31(2):339–59.

12. Fraser K, Ma I, Teteris E, Baxter H, Wright B, Mclaughlin K. Emotion, cognitive load and learning outcomes during simulation training. Med Educ. 2012;46(11):1055–62.

13. Scharfstein LA, Beidel DC, Sims VK, Rendon Finnell L. Social skills deficits and vocal characteristics of children with social phobia or asperger’s disorder: A comparative study. J Abnorm Child Psychol. 2011;39(6):865–75.
